# The bZIP transcription factor FpAda1 is essential for fungal growth and conidiation in *Fusarium pseudograminearum*

**DOI:** 10.1007/s00294-019-01042-1

**Published:** 2019-11-06

**Authors:** Linlin Chen, Yuming Ma, Jingya Zhao, Xuejing Geng, Wenbo Chen, Shengli Ding, Haiyang Li, Honglian Li

**Affiliations:** 1 College of Plant Protection, Henan Agricultural University, Zhengzhou, 450000 China; 2 National Key Laboratory of Wheat and Maize Crop Science, Zhengzhou, 450000 China

**Keywords:** *Fusarium pseudograminearum*, Transcription factors, FpAda1, Fungal growth, Cell cycle

## Abstract

*Fusarium pseudograminearum* is an important pathogen of *Fusarium* crown rot and *Fusarium* head blight, which is able to infect wheat and barley worldwide, causing great economic losses. Transcription factors (TFs) of the basic leucine zipper (bZIP) protein family control important processes in all eukaryotes. In this study, we identified a gene, designated *FpAda1*, encoding a bZIP TF in *F*. *pseudograminearum*. The homolog of FpAda1 is also known to affect hyphal growth in *Neurospora crassa*. Deletion of *FpAda1* in *F*. *pseudograminearum* resulted in defects in hyphal growth, mycelial branching and conidia formation. Pathogenicity assays showed that virulence of the *Δfpada1* mutant was dramatically decreased on wheat coleoptiles and barley leaves. However, wheat coleoptile inoculation assay showed that *Δfpada1* could penetrate and proliferate in wheat cells. Moreover, the FpAda1 was required for abnormal nuclear morphology in conidia and transcription of *FpCdc2* and *FpCdc42*. Taken together, these results indicate that FpAda1 is an important transcription factor involved in growth and development in *F*. *pseudograminearum*.

**Electronic supplementary material:**

The online version of this article (10.1007/s00294-019-01042-1) contains supplementary material, which is available to authorized users.

## Introduction

The plant pathogen *Fusarium pseudograminearum* is the causative agent of *Fusarium* crown rot (FCR) in wheat and barley, resulting in substantial yield losses worldwide (Kazan and Gardiner 2018). Particularly, in the Huanghuai wheat-growing region of China, it has been reported that *F*. *pseudograminearum* was the dominant pathogen of FCR (Li et al. 2012; Zhou et al. 2019). *F*. *pseudograminearum* was initially recognized as a population within the *Fusarium graminearum* species group (group 1). However, *F*. *pseudograminearum* is heterothallic and it was segregated by molecular analyses (Aoki and O’Donnell 1999; Gardiner et al. 2018). Like *F*. *graminearum*, *F*. *pseudograminearum* also causes *Fusarium* head blight (FHB) and produces deoxynivalenol (DON) mycotoxin under favorable conditions (Kazan and Gardiner 2018; Obanor et al. 2013). Despite the devastating effects caused by FCR and FHB, establishing effective disease management strategies has been very difficult. Therefore, understanding the molecular mechanism of pathogenicity in *F*. *pseudograminearum* is of utmost relevance, given its value in the design of a proper strategy for FCR and FHB disease management.

Transcription factors (TFs) are DNA-binding proteins that interact with other components of the transcriptional machinery to regulate the expression of multiple genes. TFs can be classified into several categories based on primary and/or three-dimensional structure similarities in the DNA-binding and multimerization domains (Riechmann et al. 2000; Warren 2002). The family of transcription factors containing a basic leucine zipper domain (bZIP) is widely distributed across eukaryotes (Hurst 1995; Kong et al. 2015). In plants, bZIP proteins are the largest protein family, which regulate processes including abiotic stress, seed maturation, flower development and pathogen defense (Alves et al. 2013; Amorim et al. 2017). In *Saccharomyces cerevisiae*, the bZIP TF family contains 14 genes, and the largest is the YAP1 group, formed by eight members. Five YAP1 family members (YAPs 1, 2, 4, 5 and 6) have been implicated in oxidative stress and DNA-damage responses (He and Fassler 2005; Tan et al. 2008; Workman et al. 2006). Filamentous fungi typically contain well over a dozen of these TFs. Several fungal bZIPs have been characterized and implicated in multiple phenomena including remediation of development, amino acid biosynthesis, unfolded protein response, nutrient utilization and various stress responses (Guo et al. 2010; Kong et al. 2015; Son et al. 2011). The bZIP protein AP-1 is essential for pathogens’ growth, development, infection and pathogenicity in *Magnaporthe oryzae*, *Ustilago maydis* and *Colletotrichum gloeosporioides*, among others (Guo et al. 2011; Li et al. 2017; Molina and Kahmann, 2007). In *N*. *crassa*, out of nine characterized bZIP members, Ada-1 (all development altered-1) is unique by regulating growth under minimal media conditions (Colot et al. 2006; Tian et al. 2011). In *F*. *graminearum*, a total of 22 bZIP TFs were functionally analyzed, and six TFs were associated with growth and pathogenicity. Among these, deletion of an *Ada*-*1* homolog (*GzbZIP001*) resulted in growth and virulence defects in *F*. *graminearum* (Son et al. 2011). However, there has been no research on Ada-1-like transcription factor in *F*. *pseudograminearum*, and their regulatory mechanism is not clear.

Cell cycle regulation is pivotal for proper cell division and cellular differentiation in eukaryotic cells. The central regulators that govern eukaryotic cell cycle progression are cyclin-dependent kinases and their partners (Bloom and Cross 2007; Humphrey and Pearce 2005; Sendinc et al. 2015). In model organisms such as yeast and *N*. *crassa*, Cdc2 is essential for cell cycle progression and hyphal growth (Booher and Beach 1986; Borkovich et al. 2004). *F*. *graminearum* has two *Cdc2* genes, *Cdc2A* and *Cdc2B*. The two Cdc2 orthologs have reproduction functions in hyphal growth and asexual reproduction, and only Cdc2A is important for plant infection and sexual reproduction (Jiang et al. 2016; Liu et al. 2015). Cdc42p is a Rho family GTPase, required for changes in polarized growth during mating and pseudohyphal development in *S*. *cerevisiae*. Cdc42p homologs in higher organisms are also associated with changes in cell shape and polarity (Moran et al. 2019; Rincon et al. 2014). The Cdc42 homolog has also been found in many fungi strains, and is required for hyphal growth (Bassilana et al. 2005; Boyce et al. 2001).

In this study, we identified FpAda1 as a homolog of the bZIP transcription factor Ada-1 in *F*. *pseudograminearum*, which was found to be involved in hyphal growth, conidiation and pathogenicity. Nuclear formation and the expression of cyclin-dependent protein kinase genes in *Δfpada1* was also studied to understand its possible regulatory network.

## Materials and methods

### Sequence analysis of FpAda1

The *Ada*-*1* (all development altered-1) gene (locus *NCU00499*) of *N*. *crassa* was downloaded from NCBI and used as the query to search against the *F*. *pseudograminearum* genome by BlastP and tBlastN algorithms (Altschul et al. 1990; Gardiner et al. 2018). The b-ZIP domain of FpAda1 was predicted by SMART (http://smart.emblheidelberg.de).

### qRT-PCR analyses

For total RNA extraction, conidia were induced in CMC medium at 150 rpm, 25 °C in the dark for 4 days. Mycelia were obtained by cultivating conidia with YEPD liquid medium at 25 °C, 150 rpm for 12 h and were then harvested by filtration over two layers of miracloth and washed with sterilized water. For conidial infection (IF18 h to IF7 days), wheat cultivar *Aikang 58*, which is susceptible to *F*. *pseudograminearum*, was grown in a greenhouse at 25 °C for 4 days. Two milliliters of conidia suspension (1 × 10^7^/ml) was infected on each coleoptile of wheat seedlings. After 18 h, 30 h, 2 days, 3 days, 5 days and 7 days’ incubation in dark at 25 °C, lesion areas with 5 mm extension were harvested. Total RNA was extracted from each sample with the RNAsimple Total RNA Kit (Tiangen, China) according to the manufacturer’s protocol. RNA was further purified and cDNA was synthesized using PrimeScript™ RT reagent Kit with gDNA Eraser (Takara, China).

The expression levels of *FpAda1* and tested *FpCdc2*, *FpCdc25*, *FpCdc42* and *FpBub1* were determined by quantitative real-time PCR (qRT-PCR) using the primers listed in Supplementary Table S1. For each sample, the *FpTEF1* gene was used as an internal control, and the following conditions were used for the qRT-PCR reaction: 95 °C for 30 s, 40 cycles at 95 °C for 5 s and 60 °C for 31 s to calculate cycle threshold values, followed by a dissociation program of 95 °C for 15 s, 60 °C for 1 min, and 95 °C for 15 s to obtain melt curves. The transcript levels of test genes were determined according to the function Δ*C*_T_ = *C*_T_ (test gene)—*C*_T_ (reference gene). To compare untreated and treated expression levels, the function ΔΔ*C*_T_ was determined using the equation ΔΔ*C*_T_ = Δ*C*_T_ (treatment) − Δ*C*_T_ (control) where the control was mock-treated with *F*. *pseudograminearum* mycelia. The induction ratio of treatment/control was then calculated 2^−ΔΔ*C*T^.

### Generation of the *FpAda1* deletion mutant and complementation strains

The split-marker approach was used to generate gene-replacement constructs for the *FpAda1* gene as described in our previous study (Chen et al. 2019a). Primers are listed in Supplementary Table S1 and a schematic diagram of primers located for gene replacement with split-marker strategy and screening of mutant is shown in Fig. 2a. Briefly, the 1147-bp upstream and 1125-bp downstream flanking sequences were amplified with primer pairs F1/R1 and F2/R2, respectively. The *hygromycin* gene (*hph*) was amplified from *pkov21* with primer pairs HYG/F and HYG/R. After three PCR cycles, a 1911-bp fusion PCR product including 5′-flanking region and 5′-*hph* region was obtained by overlap PCR amplification with primer pair A1 + HY/R using mixed fragments of *FpAda1* upstream and *hph* fragments as templates. At the same time, a 2188-bp fusion PCR product including 3′-*hph* region and 3′-flanking region was obtained by overlap PCR amplification with primer pair YG/F + B2 using mixed fragments of *FpAda1* downstream and *hph* fragments as templates. Products obtained by the third PCR cycle were used for fungal transformation. Putative gene deletion mutants were identified by PCR assays using the primers G1/G2, H2F/H2R, F3/H1R and H1F/R3. Genome DNA was digested by *EcoR* I and separated by agarose gel electrophoresis. The *hygromycin* gene was detected by the DIG DNA Labeling and Detection Kit (Roche, USA) according to the manufacturer’s protocol.

The plasmid pYIP-102 was used for construction of the complementation vector. The *FpAda1* gene with its native promoter was amplified using primers ComF/ComR and inserted into the vector. The constructed vector was transformed into a *Δfpada1* mutant. The complemented transformants were confirmed by western blot analysis.

### Phenotype determination

For mycelial growth assays, 5-mm mycelial plugs were taken from the edge of a 3-day-old colony of each strain and placed on PDA plates and incubated at 25 °C. Mycelial morphology was observed 12 h later, and colony diameters were measured and photographed 3 days later. For the conidiation assay, two 5-mm plugs from the edge of a 3-day-old colony of each strain were inoculated in 100 ml CMC. After 4 days’ cultivation in a 150-rpm-shaker at 25 °C, conidia were harvested by filtering through a layer of miracloth and counted using a hemocytometer. For the conidia germination assay, 0.1 ml of 10^4^ conidia/ml suspension was prepared and cultured in sterile distilled water at 25 °C in the dark for 3 h and 6 h. Three biological replicates were used for each strain and each experiment was repeated three times independently. Data were analyzed using a Student’s *t* test. To probe for nuclei, 2 µM DAPI dilactate (Takara, China) was used.

### Pathogenicity assays

For virulence on wheat coleoptiles, 5-mm mycelial plugs from the edge of a 3-day-old PDA plate of each strain were inoculated onto the wheat coleoptiles of the susceptible cultivar *Aikang 58*. The fungal discs were removed after 24 h, and seedling lesion lengths were photographed at 3 days post-inoculation (dpi). All experiments were performed three times with five replicates per experiment. For virulence on malting barley leaves, barley seeds were planted in pots for 14 days, and then 5-mm mycelial plugs from the edge of a 3-day-old PDA plate of each strain were inoculated onto the barley leaves. All experiments were performed three times with five replicates per experiment. For pot-culture experiments, susceptible wheat cultivar *Aikang 58* plants were planted in sterile soil mixing 0.5% inoculation millet for 10 days. Then wheat growth and infection were analyzed and documented. For observation of mycelium growth in infected coleoptiles, the inoculated wheat coleoptiles were harvested after 30 h, and epidermal cells were viewed under a Nikon *Ti*-*s* instrument.

## Results

### Identification and expression of *FpAda1* in *F*. *pseudograminearum*

**Fig. 1:**
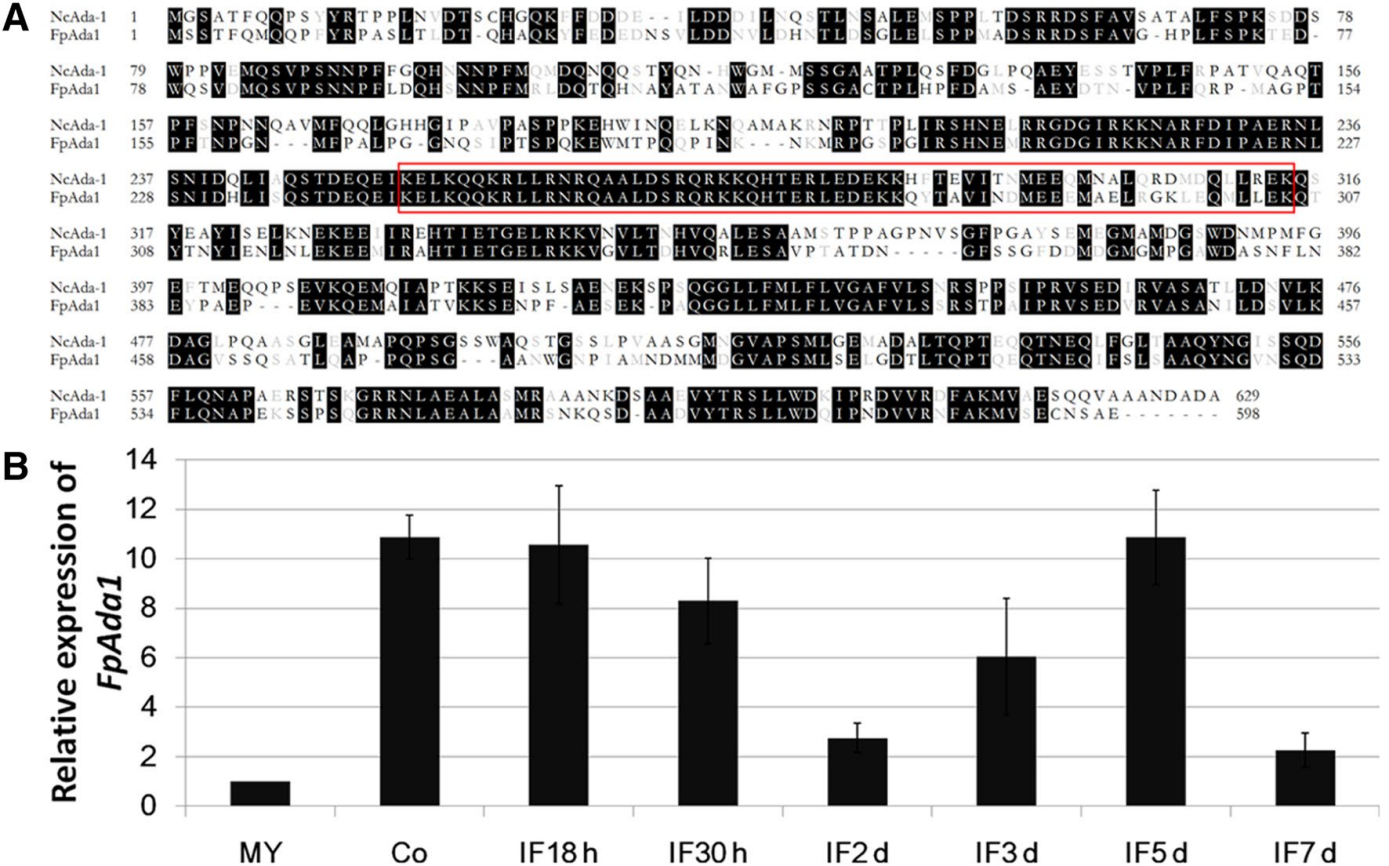
Sequence alignment and expression profiles of *FpAda1*. **a** Sequence alignment of the predicted amino acid sequence of FpAda1 with its ortholog from *Neurospora crassa* (NcAda-1). The red box indicates the b-ZIP domain. **b** Expression of *FpAda1* in hyphae, conidia, and infected wheat coleoptiles from 18 h to 7 days post fertilization

One putative all development altered-1 gene (*FPSE_04421*, designated as *FpAda1*) in *F*. *pseudograminearum* was retrieved by BLAST search of the *F*. *pseudograminearum* genome with the *N*. *crassa Ada*-*1* (*NCU00499*) as a query. The *FpAda1* gene is predicted to encode a 598-amino acid protein showing 61% identity match to *N*. *crassa Ada*-*1*. The domain analysis showed that FpAda1 has a conserved bZIP DNA-binding domain (Fig. 1a).

To further investigate the potential functions of *FpAda1* gene during development and pathogenicity in *F*. *pseudograminearum*, total RNA samples of mycelia, conidia and conidial infection wheat plants (IF18 h to IF7 days) were obtained. By qRT-PCR we observed that *FpAda1* expression was induced during conidiation and early infection stages (IF18 h and IF30 h), and a high transcriptional level of *FpAda1* was also detected at IF5 days (Fig. 1b). These results indicate that FpAda1 might play roles in both conidiation and virulence.

### Deletion and complementation of *FpAda1* gene in *F*. *pseudograminearum*

**Fig. 2:**
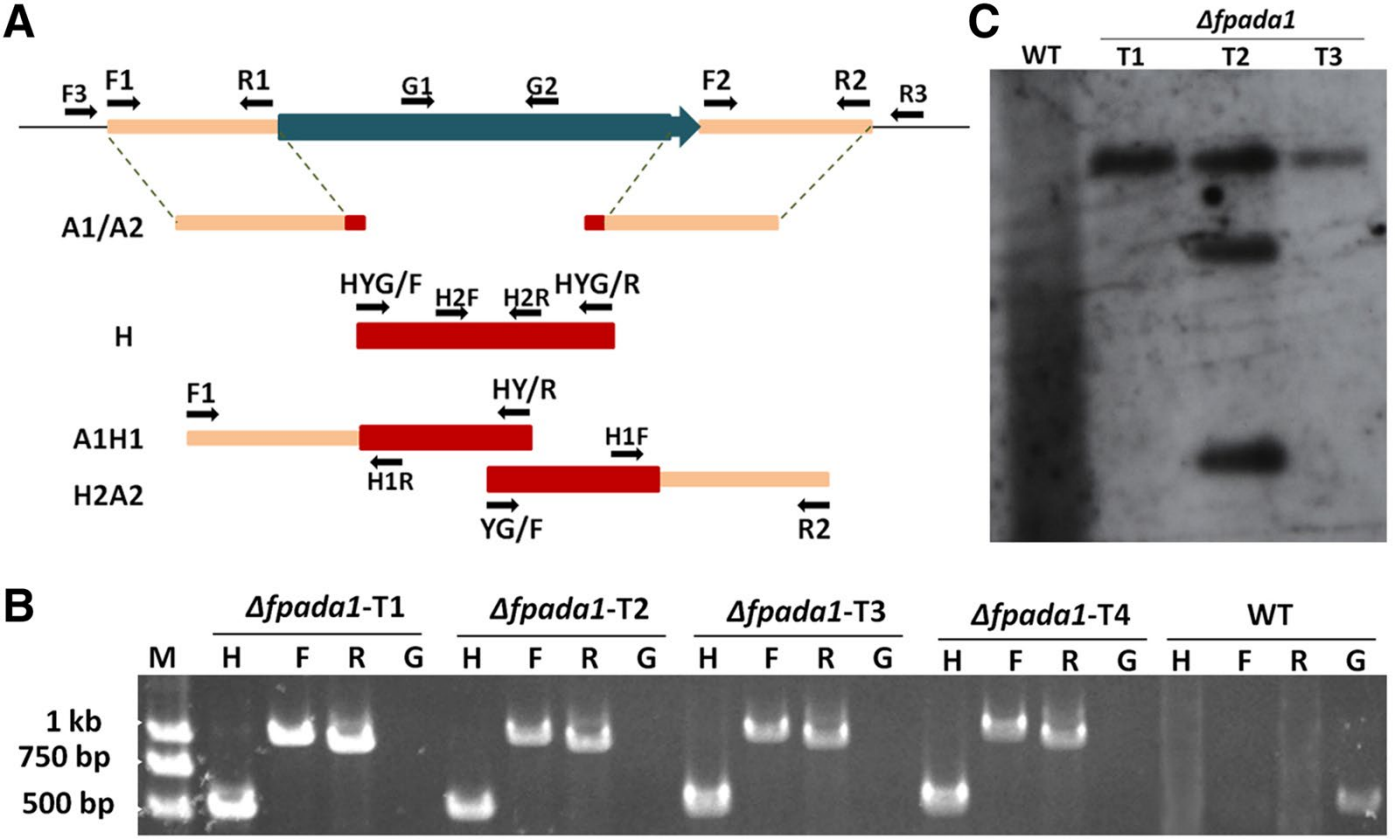
Generation and identification of *FpAda1* gene deletion mutant. **a** Gene deletion strategy for *FpAda1*. Primers used for gene replacement and screening of mutant are indicated by arrows. **b** Confirmation of *FpAda1* deletion mutants by PCR strategy. Verification of incorporation into genomic DNA by PCR using four pairs of primers, which were used to analyze *hygromycin* (H2F/H2R), upstream (F3/H1R), downstream (H1F/R3) and the *FpAda1* gene (G1/G2) positivity. Amplified fragments were 523, 991, 939 and 511-bp long. *WT* wild-type strain *WZ2*-*8*, *M* molecular markers, *H hygromycin* gene, *F* upstream, *R* downstream, *G FpAda1* gene. **c** Southern blot analysis of WT and *Δfpada1* using the 750-bp DNA fragment of *hygromycin* as a probe. The genomic DNA preparation of each strain was digested with *EcoR* I

To determine the biological function of *FpAda1* gene in *F*. *pseudograminearum*, *FpAda1* deletion mutants were generated. In Fig. 2a, a schematic diagram shows the strategy that was used to generate *FpAda1* gene deletion mutants and molecular verification of *Δfpada1*. Transformants were selected on hygromycin-amended medium, and four individual targeted deletion mutants, designated *Δfpada1*-T1, *Δfpada1*-T2, *Δfpada1*-T3 and *Δfpada1*-T4, were created and checked by PCR (Fig. 2b). However, due to bacterial contaminations, *Δfpada1*-T4 was discarded. Finally, *Δfpada1*-T1 and *Δfpada1*-T3 were confirmed as *FpAda1* gene knock-out transformants by southern blot analysis (Fig. 2c). In order to confirm that phenotypic defects in mutants were caused by *FpAda1* gene deletion, we complemented the mutant with a wild-type *FpAda1* gene with its native promoter, and a FLAG-tag was fused to the C-terminal of FpAda1. We confirmed the complemented strain *Δfpada1*-C by western blot (Fig. S1).

### FpAda1 is critical for vegetative growth in *F*. *pseudograminearum*

**Fig. 3:**
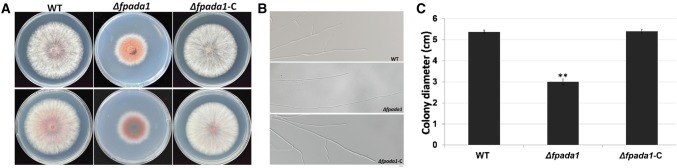
Hyphal growth and pigment formation of the *Δfpada1* mutant. **a** The WT, *Δfpada1* mutants and the complemented strain *Δfpada1*-*C* were grown on PDA plates for 3 days. **b** Colony diameters were assayed. Linear bars in each column denote standard errors of three experiments. Two asterisks indicate significant difference of colony diameter (*P* < 0.01). **c** Hyphal tip growth and branching patterns of *F*. *pseudograminearum* grown on PDA medium for 12 h. Bars = 20 μm

To evaluate the influence of FpAda1 in the vegetative growth of *F*. *pseudograminearum*, we examined the growth of *Δfpada1* cultured on PDA medium for 3 days. Growth assessment records showed that *FpAda1* deletion caused a significant reduction in the strain’s vegetative growth (Fig. 3a, c). In comparison with the wild-type and *Δfpada1*-C strains, colony pigment deposition increased in *Δfpada1* (Fig. 3a). Further microscopic examination showed that the hyphae from the *Δfpada1* mutant were thinner and produced less branches at the hyphal tip as compared with the growing hyphae of the wild-type and the complemented strains (Fig. 3b). Thus, FpAda1 played an important role in the growth and hyphal branching in *F*. *pseudograminearum*.

### FpAda1 is important for conidiation in *F*. *pseudograminearum*

**Fig. 4:**
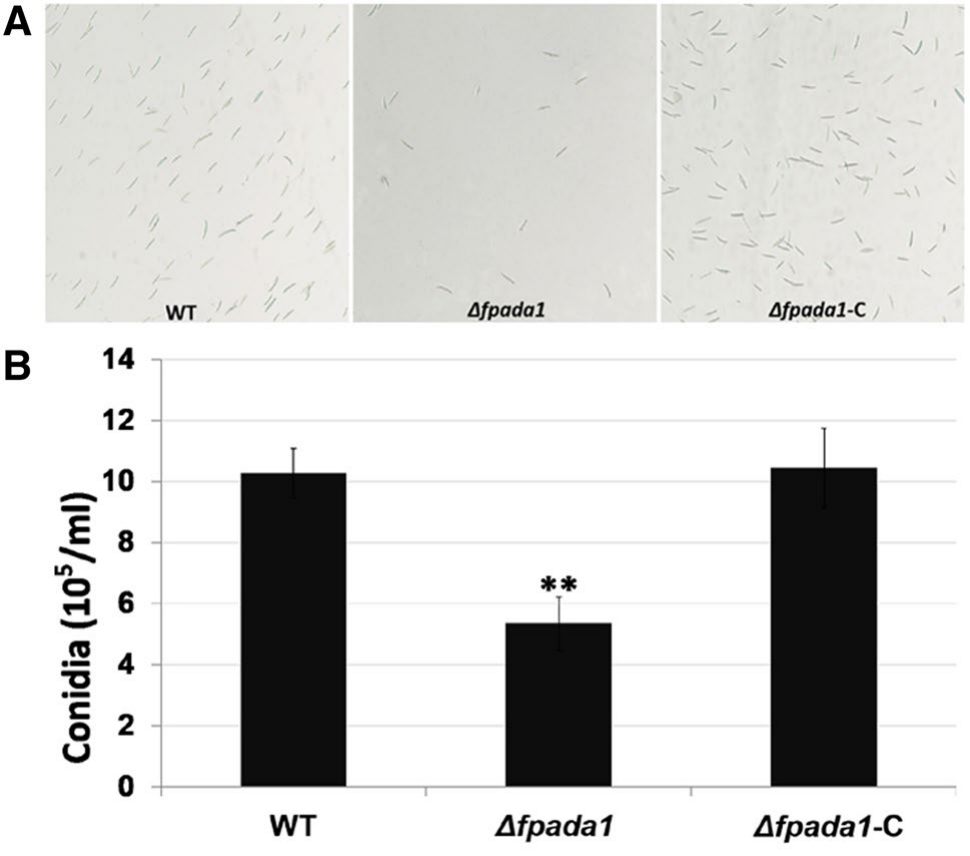
Conidial production of the *Δfpada1* mutant. **a** Conidial production (4 days after incubation in liquid CMC) of WT, *Δfpada1* mutants and the complemented strain *Δfpada1*-*C* were examined by microscopy. **b** Number of conidia produced by each line was measured at 4 dai. Data shown are representative of three separate experiments. The bars indicate standard error. **P < 0.01 (*t* test)

We performed conidial production test in *F*. *pseudograminearum* to study the function of FpAda1. Cultures of the *Δfpada1* mutant produced few conidia on CMC medium when compared with wild-type and *Δfpada1*-C strains (Fig. 4a). After 4 days of incubation, only 5.36 ± 0.88 × 10^5^ conidia/ml were obtained from the *Δfpada1* mutant, in contrast to 10.27 ± 0.81 × 10^5^ and 10.45 ± 1.30 × 10^5^ conidia/ml in WT and *Δfpada1*-C strains, respectively (Fig. 4a). These results indicate that FgAda1 is important for conidia production in *F*. *pseudograminearum*.

**Fig. 5:**
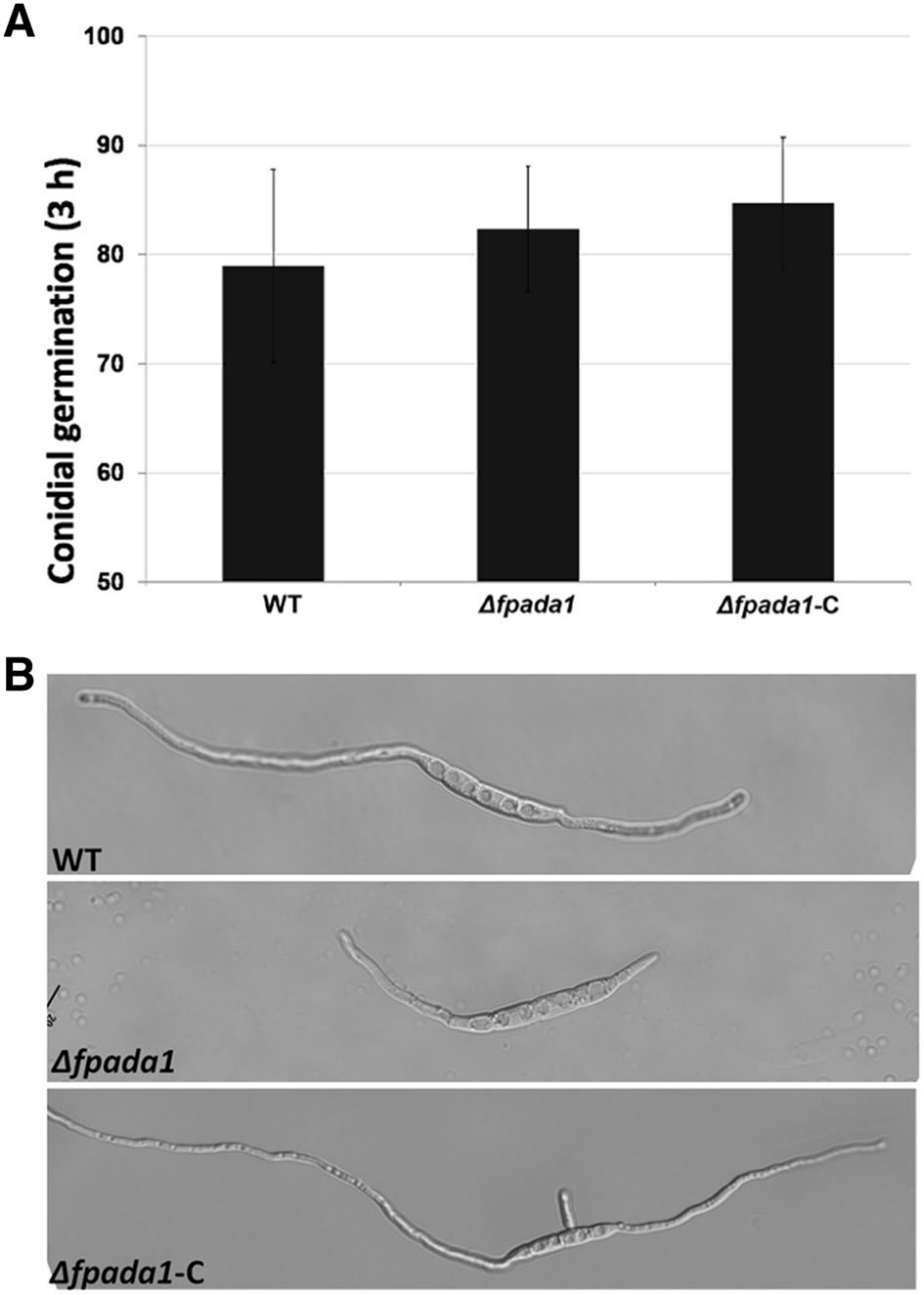
Conidial germination of the *Δfpada1* mutant. **a** Conidia germination rates were measured at 3 h after incubation in three independent biological replicates, each of which comprised of at least five glass slides. The bars indicate standard errors. **b** Conidia germlings (6 h after incubation in water) were examined by microscopy. Bars = 20 μm

To further study the function of FpAda1 in the development of conidiation, we monitored conidia germination in the *Δfpada1* mutant. The conidia collected from WT, *Δfpada1* and *Δfpada1*-C were incubated in sterile distilled water. After 3 h, the conidia in the mutant were able to germinate and we saw a synchronization compared to the wild-type and *Δfpada1*-C strains (Fig. 5a). However, the tube length of *Δfpada1* was obviously shorter than that of WT and *Δfpada1*-C strains after 6 h (Fig. 5b), which might be a consequence of a reduction in growth rate (Fig. 3a). These results indicated that FpAda1 played important roles in growth and conidiation, but not in conidia germination.

### FpAda1 affects the pathogenicity in *F*. *pseudograminearum*

**Fig. 6:**
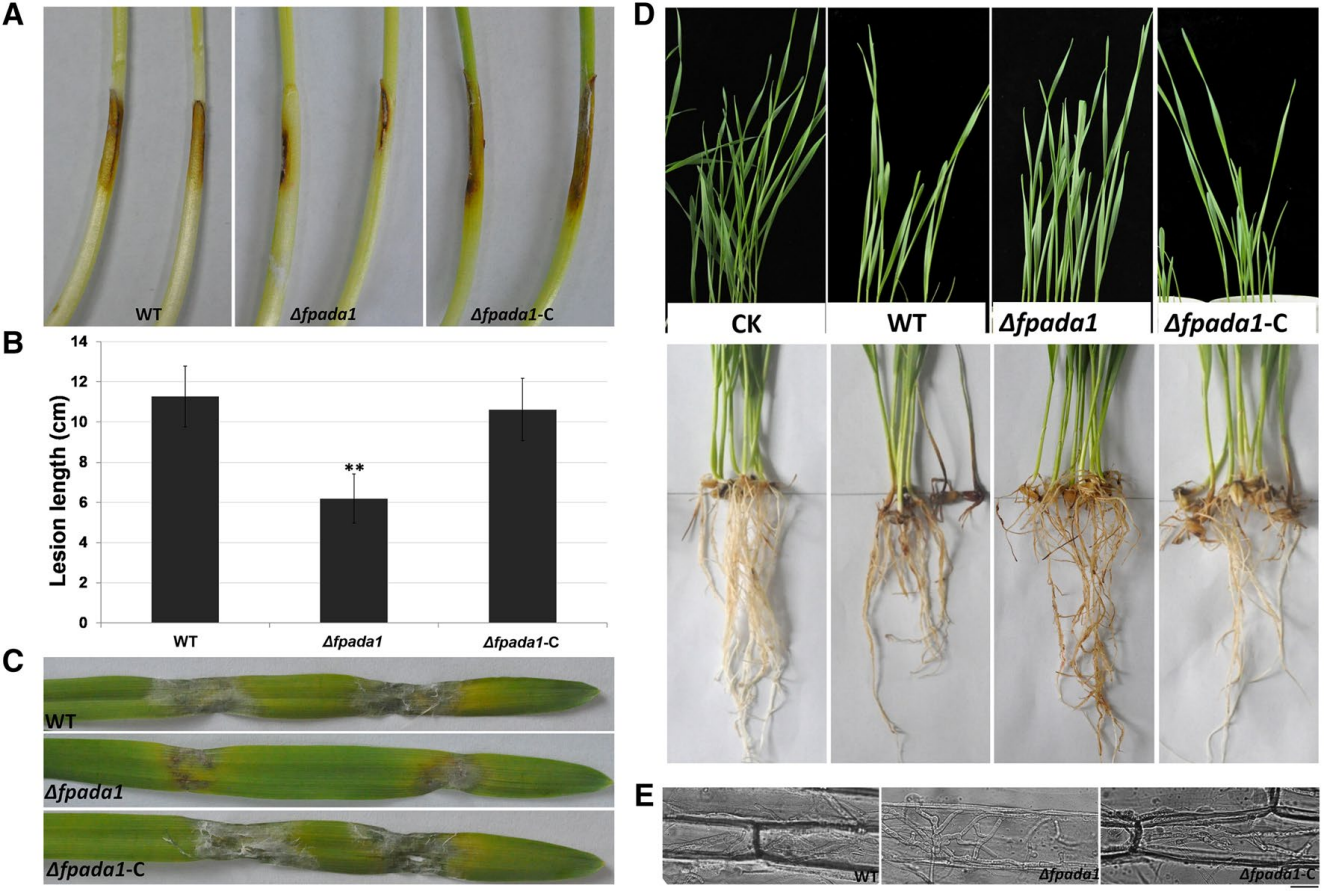
Pathogenicity assays of the *Δfpada1* mutant. **a** Wheat seedling hypocotyls were inoculated with mycelial plugs of WT, *Δfpada1* mutants and the complemented strain *Δfpada1*-*C* and examined 3 days post-inoculation. **b** Lesion lengths on wheat hypocotyls were measured at 3 days post-inoculation. The bars indicate the standard errors. **P < 0.01 (*t* test). **c** Barley leaves were inoculated with mycelial plugs and examined 5 days post-inoculation. **d** Wheat growth and wheat root lesions were examined from the pot-culture experiment at 10 days post-inoculation. **e** Infection mycelia in wheat hypocotyl cells were examined at 30 h post-inoculation. Bars = 20 μm

To investigate the role of FpAda1 in fungal virulence, we first inoculated wheat coleoptiles with WT, *Δfpada1* and *Δfpada1*-C strains. The average length of brown lesions on the wheat coleoptiles infected with the *Δfpada1* mutant was 0.62 ± 0.12 cm, whereas those infected with the WT and *Δfpada1*-C strains showed average lesion lengths of 1.13 ± 0.15 and 1.06 ± 0.16 cm, respectively (Fig. 6a, b). Furthermore, we also inoculated the aforementioned strains on barley leaves. The *Δfpada1* mutant also caused lesions at the inoculated leaves, but the mutant-caused disease effects were less pronounced as compared with the WT and *Δfpada1*-C under the same conditions (Fig. 6c). Finally, a pot-culture experiment was used to further confirm the involvement of FpAda1 in fungal virulence. The WT and *Δfpada1*-C caused crown rot symptoms in wheats at 10 days post inoculation (dpi). However, wheat seedlings showed mild symptoms after inoculation with the *Δfpada1* mutant (Fig. 6d).

We then evaluated the effects of *Δfpada1* mutant on the fungal invasion process in wheat at a cellular level. After inoculation of wheat coleoptiles, microscopic analysis showed that hyphae of WT, *Δfpada1* and *Δfpada1*-C infected and extended similarly in coleoptile cells (Fig. 6e). The results suggest that the observed reduction in virulence might be a consequence of a reduction in growth rate of *Δfpada1* mutant.

### FpAda1 is involved in the cell cycle of *F*. *pseudograminearum*

**Fig. 7:**
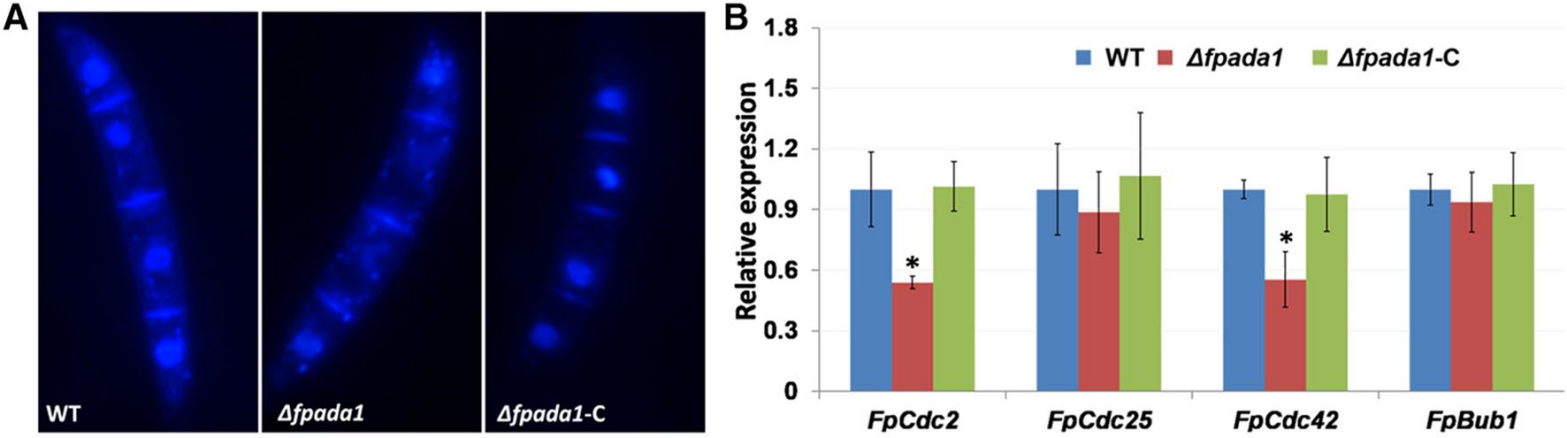
a Nuclei of WT, *Δfpada1* mutants and the complemented strain *Δfpada1*-C were stained with DAPI and images were taken. Bars = 10 μm. **b** Relative transcription levels of cyclin-dependent protein kinase genes (*FpCdc2*, *FpCdc25*, *FpCdc42*) and serine/threonine protein kinase (*FpBub1*) gene in WT, *Δfpada1* mutant and the complemented strain *Δfpada1*-C. For each gene, the expression level in WT was arbitrarily set as 1. Bars denote standard errors from three repeated experiments. *P < 0.05 (*t* test)

Cell cycle regulation has been shown to be important for growth and morphological changes. Because *Δfpada1* exhibited severe defects in growth and development, we assessed the expression levels of nucleus and cell division cycle genes. DAPI staining revealed that the *Δfpada1* cells had abnormal nuclear morphology in conidia (Fig. 7a). In WT and *Δfpada1*-C strains, one disc-shaped nucleus could be observed in every cell. However, nuclei-lacking cells were widespread in *Δfpada1* conidia. In addition, the expression of three cyclin-dependent protein kinase genes (*FpCdc2*, *FpCdc25* and *FpCdc42*), involved in fungal growth, were analyzed in WT, *Δfpada1* and *Δfpada1*-C strains. A serine/threonine protein kinase (*FpBub1*) was chosen as contrast for the cyclin-dependent protein kinase genes. The expression levels of *FpCdc2* and *FpCdc42* were significantly reduced in *Δfpada1* mutant (Fig. 7b), which further supported the role of FpAda1 in cell cycle regulation.

## Discussion

The bZIP transcription factors have been reported to regulate many central physiological and developmental processes in plants, such as flowering, seed maturation, stress response and pathogen defense (Alves et al. 2013; Banerjee and Roychoudhury 2017). Recently, a number of bZIP transcription factors have been identified in plant pathogenic fungi and played important roles in development, stress response and virulence. In *M*. *oryzae*, 22 bZIP transcription factors were identified and characterized as being involved in development, nutrient utilization and various stress responses (Kong et al. 2015). For example, MpAtf1 regulated the transcription of laccases and peroxidases, which was critical in pathogenicity (Guo et al. 2010). In *F*. *graminearum*, transcription factors related to growth, development, stress responses and virulence were reported (Chen et al. 2019b; Lv et al. 2019). However, few transcription factors have been described in *F*. *pseudograminearum*.

In this study, we have characterized a bZIP-type transcription factor FpAda1 in *F*. *pseudograminearum* as a homolog of *N*. *crassa* Ada-1 protein. Similar to other bZIP proteins, FpAda1 contains a bZIP DNA-binding domain. In *N*. *crassa*, the bZIP TF family contains nine genes and can be divided into two groups. Ada-1 was clustered to the GCN4 clade, and the *Δada*-*1* mutant showed a reduced growth rate and very short aerial hyphae. Among the strain carrying a deletion of the *bZIP* gene, *Δada*-*1* showed the greatest number of expression differences from the WT with 290 genes increasing, and 219 genes decreasing, consistent with its growth defect (Tian et al. 2011).

To determine the biological function of FpAda1 in *F*. *pseudograminearum*, its deleted mutant was generated. Compared to wild type, the mutant showed defects in hyphal growth, mycelial branching and conidia formation. However, in *F*. *graminearum*, the *Ada*-*1* homolog *GzbZIP001* mutant showed no significant changes in conidiation. GzbZIP001 was required for the pathogenicity of *F*. *graminearum*, and its deleted mutant could not cause disease in wheat (Son et al. 2011). However, the *Δfpada1* mutant could infect wheat and the deficiency in pathogenicity might be due to reduction of growth.

In fungi, cell cycle regulation has been shown to be important in terms of growth and development, and this helps ensure that cells maintain their normal size, shape and nuclear number (Ahmadian et al. 2019; Jiang et al. 2016). The cyclin-dependent protein kinases CDKs are the central regulators of the eukaryotic cell cycle (Liu et al. 2015). The Cdc2 kinase in yeasts and filamentous fungi has a key regulatory role in the cell cycle. Unlike other fungi, *F*. *graminearum* have two *Cdc2* genes, and the two Cdc2 orthologs play different roles in vegetative and infectious hyphae (Sudbery 2008). Cdc42 is a member of the Rho family of GTPases, which are required for hyphal growth in many fungi (Nozaki et al. 2018; Si et al. 2016). Here, we found that the *FpAda1* deletion cells had abnormal nuclear morphology, and *FpCdc2* and *FpCdc42* were affected in expression.

In conclusion, our study demonstrated that the bZIP transcription factor plays important roles in growth, conidiation and pathogenesis in *F*. *pseudograminearum*. In addition, FpAda1 can affect cell cycle and the expression of *FpCdc2* and *FpCdc42*.

## Electronic supplementary material

Below is the link to the electronic supplementary material.

Fig. S1 Western blotting of FpAda1 from transformed mycelia. Total protein was extracted from mycelia grown in vitro. FpAda1 was detected with anti-FLAG antibody. FpAda1 protein is ~ 80 kDa

Table S1. Primers used in the study
