# Supplementary figures and images for: The bZIP transcription factor FpAda1 is essential for fungal growth and conidiation in Fusarium pseudograminearum
Source: Curr Genet. 2019 Nov 6;66(3):507–15. doi: 10.1007/s00294-019-01042-1 (PMC7198649; doi:10.1007/s00294-019-01042-1)

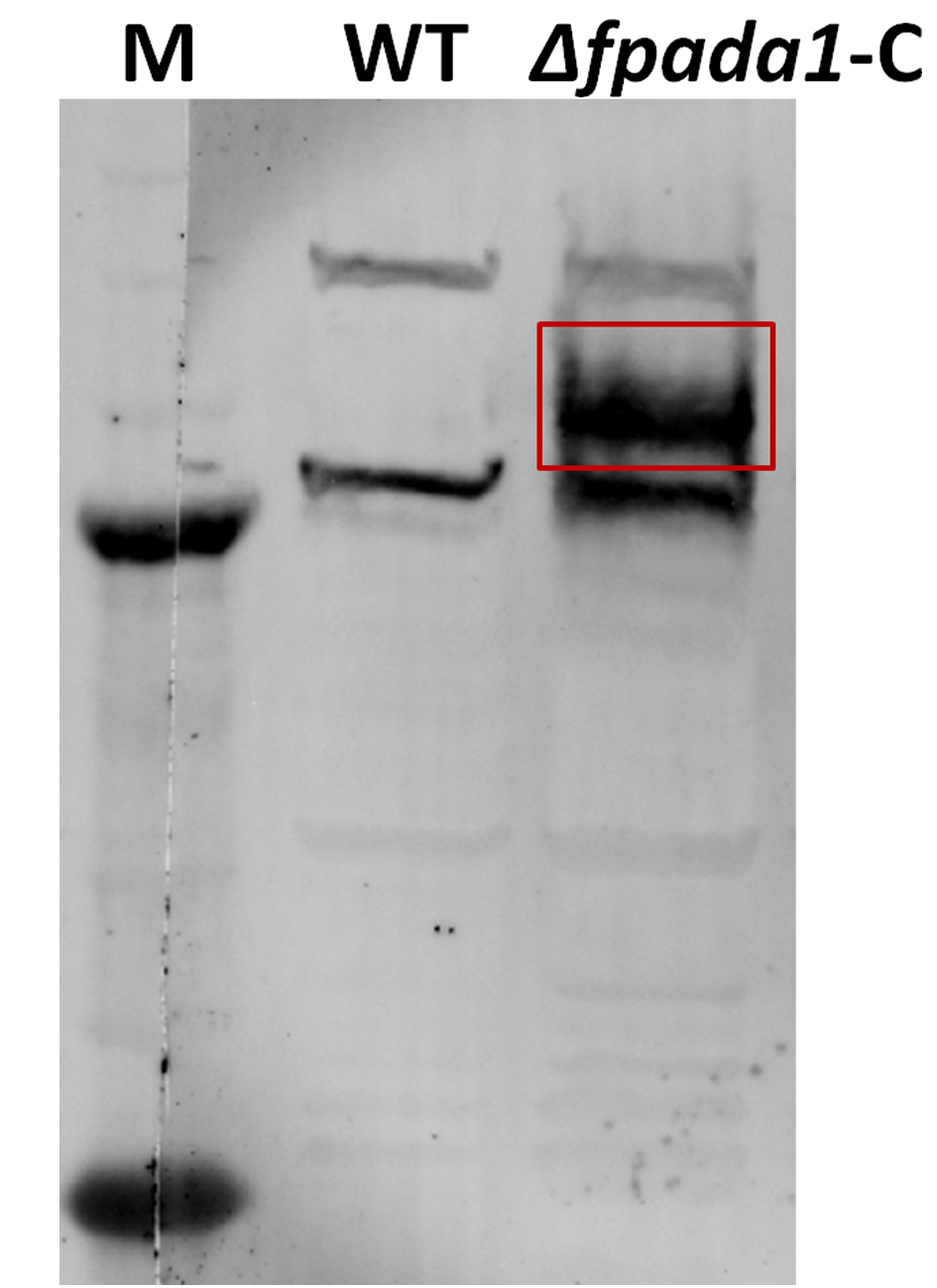

Supplement: Supplementary file 1 — Fig. S1 Western blotting of FpAda1 from transformed mycelia. Total protein was extracted from mycelia grown in vitro. FpAda1 was detected with anti-FLAG antibody. FpAda1 protein is ~ 80 kDa [file 294_2019_1042_MOESM1_ESM.jpg]
