# Supplementary material for: The bZIP transcription factor FpAda1 is essential for fungal growth and conidiation in Fusarium pseudograminearum
Source: Curr Genet. 2019 Nov 6;66(3):507–15. doi: 10.1007/s00294-019-01042-1 (PMC7198649; doi:10.1007/s00294-019-01042-1)
Supplement: Supplementary file 2 — Table S1. Primers used in the study [file 294_2019_1042_MOESM2_ESM.docx]

Table S1. Primers used in the study

| Primer | Sequence (5΄→3΄) |
| --- | --- |
| F1 | TGGATGCATTGCAGATAGAGATCC |
| R1 | CAATATCATCTTCTGTCGACTATGCAGAGTGAGTGAGGGAG |
| F2 | ATAGAGTAGATGCCGACCGCGGGTTCGCGAGCAGCAATGCAATGAAA |
| R2 | TCTTAGACAAGCCATCATCTCAGTC |
| HYG/F | GGCTTGGCTGGAGCTAGTGGAGGTCAA |
| HYG/R | GTATTGACCGATTCCTTGCGGTCCGAA |
| HY/R | GTATTGACCGATTCCTTGCGGTCCGAA |
| YG/F | GATGTAGGAGGGCGTGGATATGTCCT |
| H2F | ACGTCTGTCGAGAAGTTTCTG |
| H2R | GCATCAGCTCATCGAGAGCCT |
| G1 | AGCAACATTGACCATCTTATC |
| G2 | TGGCAATAGCCATCTCCTGC |
| F3 | TCATCATATCCTTCCCTTCTTG |
| H1R | TCACAGTTTGCCAGTGATACAC |
| H1F | TGTCACGTTGCAAGACCTGCCTG |
| R3 | AATAGCCGACTTCAAGGAAAGG |
| ComF | CGCGGGCCCCTCCACACCCACAGAGATCT |
| ComR | CCATCGATTTCTGCGCTGTTGCATTC |
| FpTEF1-RTF | TCACCACTGAAGTCAAGTCC |
| FpTEF1-RTR  FpAda1-RTF  FpAda1-RTR | ACCAGCGACGTTACCACGTC  CAGACCTACACCAACTAC  GATGAGAAGCCGTTGTCTGTG |
| FpCdc2-RTF | ACGAGGTTGTCACTCTCTGG |
| FpCdc2-RTR | ACATCTCGGCGAAGATGGTTC |
| FpCdc25-RTF | TGTTCTGAACAACTTCTCG |
| FpCdc25-RTR | TACGCTGAGGCACTTGGTC |
| FpCdc42-RTF | TCAAGAAGATTACGACCGAC |
| FpCdc42-RTR | GAACCATTTCTCGCGAACG |
| FpBub1-RTF | GAGGGTCGACCCTGGACATG |
| FpBub1-RTR | TCAATCCACCCTGATCGCAAC |
